# Prognostic significance of BRCA1 and BRCA2 methylation status in circulating cell-free DNA of Pancreatic Cancer patients

**DOI:** 10.7150/jca.93184

**Published:** 2024-03-11

**Authors:** Triantafyllia Koukaki, Ioanna Balgkouranidou, Eirini Biziota, Anastasios Karayiannakis, Helen Bolanaki, Evangelos Karamitrousis, Paul Zarogoulidis, Savas Deftereos, Charalampos Charalampidis, Aris Ioannidis, Dimitrios Matthaios, Kyriakos Amarantidis, Stylianos Kakolyris

**Affiliations:** 1 Department of Medical Oncology, Medical School, Democritus University of Thrace, Greece; 2 Department of 2nd Surgery, Medical School, Democritus University of Thrace, Greece; 3 University Medical Oncology department, Aristotle University of Thessaloniki, Papageorgiou General Hospital.; 4 Pulmonary Oncology Department, General Clinic Euromedica, Thessaloniki, Greece; 5 Radiology Department, Medical School, Democritus University of Thrace, Greece; 6 Pathology Department, University of Cyprus, Cyprus; 7 Surgery Department, Genesis Private Clinic, Thessaloniki, Greece; 8 Oncology Department, General Hospital of Rhodes, Greece

**Keywords:** BRCA1, BRCA2gene, DNA methylation, Cell-free DNA, prognosis

## Abstract

**Introduction:** Pancreatic cancer is the most fatal cancer type in the world. Its high mortality is mostly correlated to the absence of symptoms and the difficulty in early diagnosis, which in the majority of the cases occurs when the disease has already spread metastasis. Nowadays, tests that could predict early diagnosis are not available yet and the number of prognostic tests is limited. Hence, there is an urgent need for biomarkers capable of detecting early development or the rapid progression of the disease.

**Patients and Methods:** DNA methylation represents the most frequent epigenetic event among tumor suppressor genes that are involved in various carcinogenic pathways. In the recent study we have tried to evaluate, for the first time, the prognostic value of BRCA1 and BRCA2 methylation in the cell-free DNA of pancreatic cancer patients. Using methylation-specific real-time PCR we examined the methylation status of BRCA1 and BRCA2 in 55 patients with operable and 50 patients with metastatic pancreatic cancer. In the operable disease setting, BRCA1 was found to be methylated in 33/55 (63.5%) patients examined while BRCA2 was also highly methylated in 31/55 (56.3%). In the metastatic disease, BRCA1 was found to be methylated in 26/50 (52%) while BRCA2 was found methylated in 23/50 (46%).

**Results:** All control samples were negative for BRCA1 orBRCA2 promoter methylation. Patients with operable pancreatic cancer and a methylated BRCA1 and BRCA2 promoter status had a statistically significant poorer outcome as compared with patients with a non-methylated one (p=0.012 and p=0.001, respectively).

**Conclusion:** In this study plasma methylation of BRCA1 and BRCA2 represents a frequent event in both the operable as well as in the metastatic setting. BRCA1 and BRCA2 methylation was significant and correlated with decreased survival in patients with operable pancreatic cancer. A larger cohort of patients is required to further explore the potential of these findings as well as to investigate whether BRCA1/2 methylation in plasma could serve as a potential prognostic biomarker in pancreatic cancer.

## Introduction

Pancreatic cancer represents the third leading cause of cancer-related mortality around the world with a 5-year survival rate of only 10% for patients who are under treatment 1, 2. It represents an aggressive disease that could remain asymptomatic for a long period and when it is usually diagnosed the disease has already proceeded 3. When applicable, surgical resection is the golden standard for this type of cancer. Consequentially, there is an urgent need to discover novel biomarkers for early diagnosis and also capable of identifying subsets of patients that may benefit from certain chemotherapy regimens.

Understanding the pathogenesis and complexity of cancer has led us to a new era of biomarker discovery. The genetic and epigenetic basis of pancreatic cancer are the new fonts of novel molecular biomarkers. DNA methylation is the most frequent epigenetic event that usually occurs during pancreatic cancer initiation and disease progression. There is a strong relation between DNA methylation patterns of certain tumor suppressor genes and tumor progression so it is vital to better analyze their clinical impact to identify novel candidate biomarkers with prognostic/predictive potential.

During the last decade, there have been made great efforts in studying DNA methylation signatures in circulating cell-free DNA (cfDNA). cfDNA carries methylation markers that permit the identification of tissue-specific cell death 4, 5 and on the whole are more informative, sensitive, and specific than individual DNA mutations 6. Notably, blood sample collection and plasma separation is a simple and minimally invasive procedure that allows adequate follow-up under minimal stress conditions for the patient.

Many susceptibility genes including ATM, BRCA1, BRCA2, CDKN2A, MLH1, MSH2, MSH6, PALB2, PMS2, PRSS1, STK11, and others, have been related to an increased risk of pancreatic cancer 7.

BRCA1 and BRCA2 proteins play an important role in the response to and repair of DNA double-strand breaks through the homologous recombination DNA repair (HRR) pathway 8.HR pathway has the highest fidelity and precision of the double-strand brake repair pathways. As a result, defects in this pathway (homologous repair deficiency, HRD) lead to error-prone repair and genomic instability, increasing cancer risk 9.

Mutations of the BRCA1 and BRCA2 genes were first associated as breast and ovarian cancer risk factors among studies that aim to characterize the genes responsible for familial clustering of breast and ovarian cancers 10, 11. Between cohorts of pancreatic cancer patients, multiple studies estimated the incidence of germline pathogenic BRCA mutations 12, 13. Their presence is linked to the disease incidence but their effect on the clinical features of the disease is less clear. Several studies have shown different results regarding the prognostic significance. As with other cancer types concomitant somatic BRCA1 (sBRCA1) promoter methylation has been described. In particular, BRCA1 promoter is methylated in different tumor types including breast (up to 20%) and ovarian (up to 19%) sporadic cancers 14. Nevertheless, the role of epigenetic silencing of BRCA1/2 in pancreatic cancer remains controversial.

This study investigates the methylation status of BRCA1 and BRCA2 in the cell-free DNA of patients with operable and metastatic pancreatic cancer. The correlations between the cfDNA methylation status of both genes and clinicopathological factors were determined. The impact of methylation levels on the survival outcomes of the patients was also evaluated.

## Material and Methods

### Study design: patient selection

The study material consisted of 105 samples obtained from patients with early operable and metastatic pancreatic cancer. The patients were eligible for the study if they were at least 18 years old, with newly diagnosed operable or metastatic pancreatic cancer and with Eastern Cooperative Oncology Group (ECOG) performance status of 0 - 2. They should have no other known malignant neoplasm (on follow up or under treatment) or a known family history of pancreatic, breast or ovarian cancer in first degree relatives. Patients with known significant cardiovascular disease were excluded. Their clinical characteristics are shown in table 1. We examined the methylation status of BRCA1 and BRCA2 genes in the cell-free DNA circulating in plasma taken from 55 early operable pancreatic cancer patients and from 50 metastatic pancreatic cancer patients. Samples were collected at the beginning of the adjuvant and first-line therapy, respectively. All patients provided their informed consent to participate in the study which has been approved by the Scientific Committee of our Institution. Cell-free DNA circulating in plasma was isolated from these patients and 40 healthy individuals. The healthy volunteer group is a totally independent age-matched group that had not received any medical care and had no relation with the patients.

### Sample collection and isolation of cell-free DNA circulating in plasma

Peripheral blood samples were collected in EDTA tubes. Blood was centrifuged at 1500 x g for 10 min and the plasma was stored at -80C until further analysis. Cell-free DNA (cfDNA) was extracted from 1200μL of plasma using the MagCore automated nucleic acid Εxtractor (MagCore Plasma DNA Extraction kit) according to the manufacturer's instructions. The DNA concentration was determined in the Qubit fluometer (Thermo Fisher Scientific).

### Sodium bisulfite conversion

Sodium bisulfite conversion of up to 0.5 g cell-free DNA was performed using the EZ DNA Methylation Gold Kit (ZYMO Research Co., Orange, CA), according to the manufacturer's protocol. The Universal Methylated Human DNA Standard (Zymo Research Corp., USA) was used as the 100% methylated control. In each conversion reaction, the dH2O and gDNA from the 100% methylated control were used as negative and positive controls, respectively. The converted DNA was stored at -70°C until used.

### Methylation Specific PCR (Real-time MSP)

The methylation status of BRCA1 and BRCA2 in cfDNA was detected by Real-time MSP using specific primer pairs for both methylated and unmethylated promoter sequences. Both real-time MSP assays developed and used in this study have been evaluated for their specificity and sensitivity. They detect down to 0.1% of the methylated sequences in the presence of 99.9% of non-methylated. The assays are not quantitative so it hasn't been established a cut off but we report a sample as positive when an amplification signal is detected (0-40 cycles). The MSP reactions were performed in the Qiagen Rotor-Gene instrument of a total of 20μl. Human placental genomic DNA (gDNA; Sigma Aldrich) methylated in vitro with SssImethylase (NEB, Ipswich, MA) was used, after sodium bisulfite conversion, as fully methylated (100%) MSP positive control. The same unmethylated placental gDNA was used, after sodium bisulfite conversion, as a negative MSP control. This unmethylated DNA control was mixed with 100% methylated DNA for the preparation of serial dilutions of known concentrations, for the further evaluation of the analytical sensitivity.

### Statistical analysis

Statistical analysis of the data was performed using the Statistical Package for the Social Sciences (SPSS), version 21.0 (IBM, New York, USA). The methylation status of BRCA1, BRCA2and all other qualitative variables were expressed as frequencies and percentages (%). Survival rates were calculated with the Kaplan-Meier method and the statistical difference between survival curves was determined with the log-rank test.

## Results

### Early Operable pancreatic cancer (EOPC)

#### Characteristics of the study population

The study population consisted of 55 patients with operable pancreatic cancer with a median age of 67 years (range 44-85 years; mean age ± standard deviation, 65.55±7.73 years) and 74.5% of whom were males.

#### Detection and clinical evaluation of BRCA1and BRCA2 promoter methylation in early operable pancreatic cancer

##### Correlation between BRCA1 promoter methylation and tumor parameters

We examined the methylation status of BRCA1 in cell-free DNA from 55 operable pancreatic cancer patients. A high BRCA1 promoter methylation status was observed in this group of patients (33/55; 60.0%). No statistically significant correlation between patients with a methylated BRCA1 status and different patients' tumor parameters was found.

##### Correlation between BRCA2promoter methylation and tumor parameters

BRCA2 promoter was found to be methylated in 31/55 (56.3%) of the patients. The methylation status of BRCA2 was associated with low-grade pancreatic cancer (p=0. 023).

##### Survival analysis

After a median follow-up period of 60 months, 53/55 (96.3%) patients have died as a consequence of the disease progression.

##### Correlation between BRCA1 promoter methylation and survival

**Figure 1:**
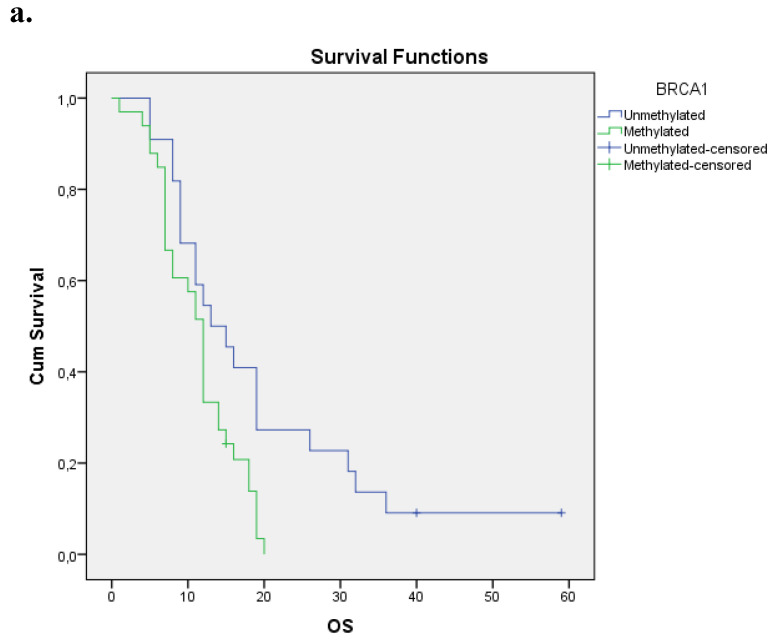

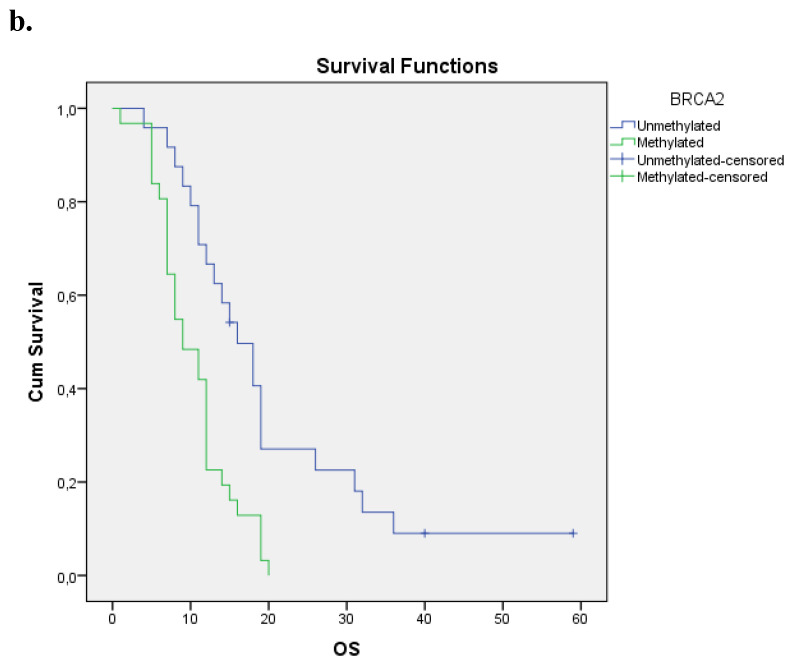

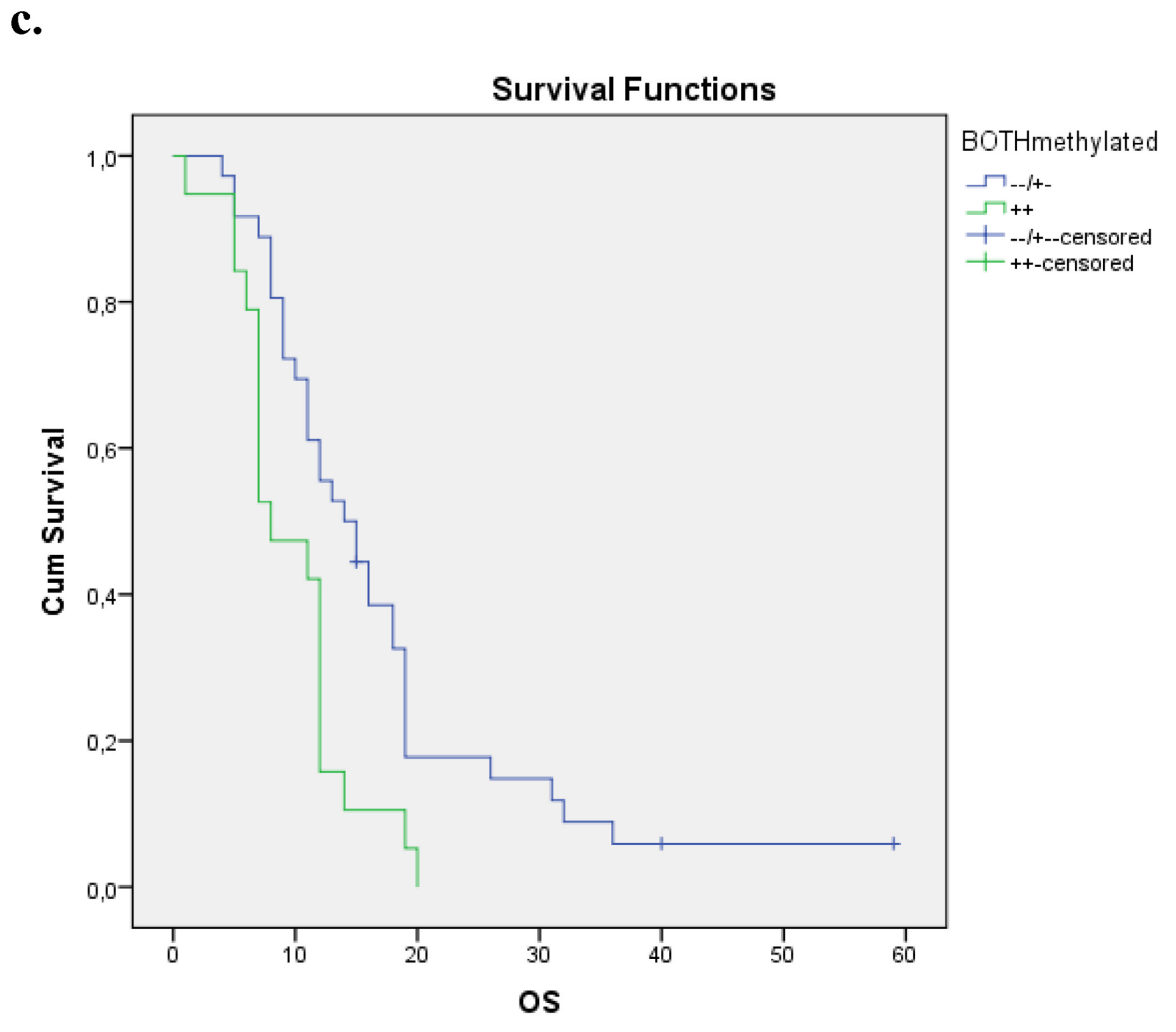
** a.** Correlation between OS and methylation status of BRCA1 in early operable pancreatic cancer, p=0.012. **b.** Correlation between OS and methylation status of BRCA2 in early operable pancreatic cancer, p=0.001. **c.** Correlation between OS and methylation status of BRCA1 and BRCA2 in early operable pancreatic cancer, p=0.004.

We evaluated the Kaplan-Meier estimates for the independent cohort of 55 patients with early operable disease, concerning the methylation status of BRCA1 promoter in cell-free DNA circulating in plasma. Patients with methylated BRCA1 gene presented a statistically significant poorer survival as compared with those having an unmethylated BRCA1 promoter status (p=0.012). Kaplan-Meier estimates of survival according to the methylation status in early-stage pancreatic cancer are given in **Figure 1a.**

##### Correlation between BRCA2 promoter methylation and survival

The Kaplan-Meier estimates for the entire cohort of patients have shown that patients with methylated BRCA2 gene presented a statistically smaller overall survival period in correlation to those with an unmethylated one (p=0.001). **Figure 1b**.

##### Correlation between both genes' promoter methylations and survival

Nineteen patients from the entire cohort presented simultaneous methylation of both genes. These patients presented a shorter overall survival compared with those with both unmethylated genes and at least one gene unmethylated p=0.004. **Figure 1c**.

### Metastatic pancreatic cancer patients (MPC)

#### Characteristics of the study population

The study population consisted of 50 patients with metastatic pancreatic cancer with a median age of 67 years (range 44-84 years; mean age ± standard deviation, 67.66±8.46 years) 58% of whom were males.

#### Detection and clinical evaluation of BRCA1 and BRCA2 promoter methylation in metastatic pancreatic cancer

##### Correlation between BRCA1 promoter methylation and tumor parameters

We examined the methylation status of BRCA1 in cell-free DNA from 50 metastatic pancreatic cancer patients. BRCA1 promoter methylation status was found in 26 out of 50 cases examined (26/50; 52%). No statistically significant correlation between patients with methylated BRCA1 status and different patients' tumor parameters was found.

##### Correlation between BRCA2 promoter methylation and tumor parameters

BRCA2 promoter was found to be methylated in 23/50 (46%) of the examined patients. The methylation status of BRCA2 was associated only with low-grade pancreatic cancer (p=0. 018).

##### Survival analysis

After a median follow-up period of 37 months, all patients have died as a consequence of the disease progression.

##### Correlation between BRCA1 promoter methylation and survival

**Figure 2:**
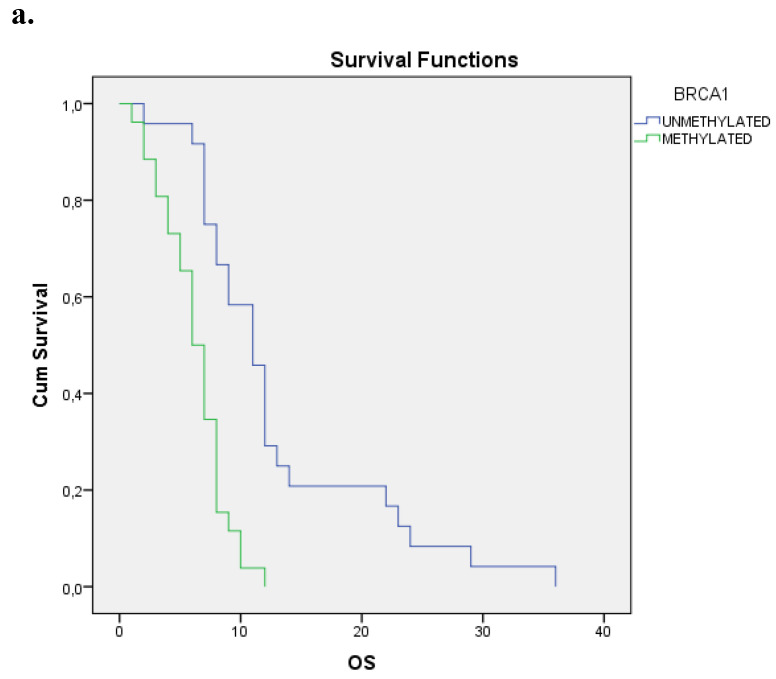

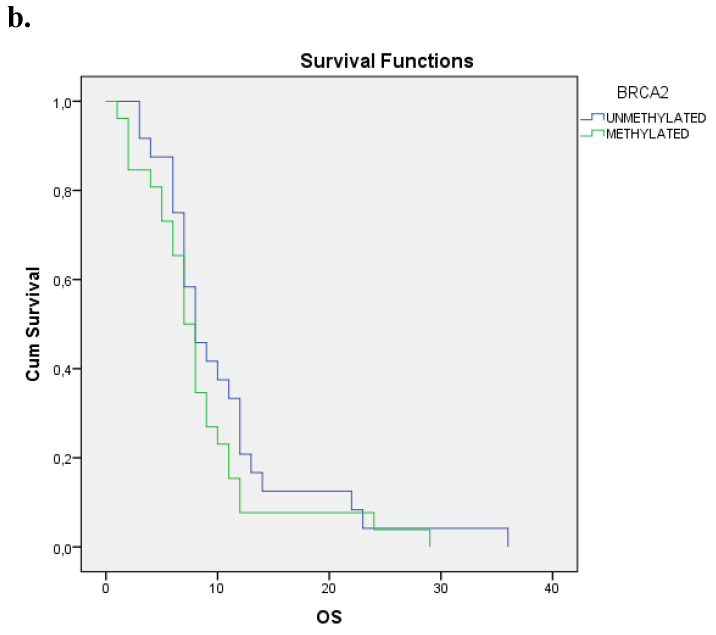

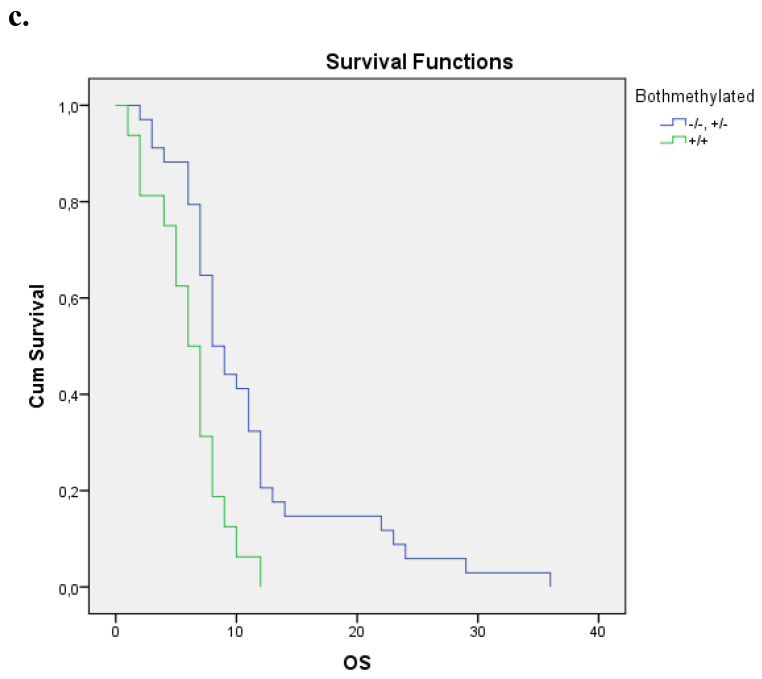
** a.** Correlation between OS and methylation status of *BRCA1* in metastatic pancreatic cancer, p=0.000. **b.** Correlation between OS and methylation status of *BRCA2* in metastatic pancreatic cancer, p=0.258. **c.** Correlation between OS and methylation status of both genes in metastatic pancreatic cancer, p=0.002.

We evaluated the Kaplan-Meier estimates for the independent cohort of 50 metastatic patients, concerning the methylation status of BRCA1 promoter in cell-free DNA circulating in plasma. Patients with methylated BRCA1 gene presented a statistically significant poorer survival as compared with those having an unmethylated one (p=0.000). Kaplan-Meier estimates of survival according to the methylation status in metastatic pancreatic cancer are given in **Figure 2a**.

##### Correlation between BRCA2 promoter methylation and survival

The Kaplan- Meier estimates for the entire cohort of patients have shown that patients with methylated BRCA2 gene have not presented a statistically significant correlation between overall survival period and methylation status (p=0.258). **Figure 2b**.

##### Correlation between both genes' promoter methylations and survival

Sixteen patients from the entire cohort presented simultaneous methylation of both genes. These patients presented a shorter overall survival compared with those with both unmethylated genes and at least one gene unmethylated p=0.002. **Figure 2c**.

## Discussion

The tumor suppressor genes BRCA1/2 and their encoded proteins are important elements of DNA double-strand break repair by homologous recombination. In genomic BRCA1/2-associated tumors, acquired mutations of the wild-type allele lead to complete loss of BRCA function and a higher sensitivity to DNA-damaging agents 8, 9. Homologous repair deficiency (HRD) leads to error-prone repair and genomic instability, increasing cancer risk.

The incidence of germline pathogenic BRCA mutations in unselected groups of pancreatic patients range from 0,2- 2,3 % for BRCA1 and 0,7-5,7 for BRCA2 carriers 13, 15.

Due to the established activity of PARP inhibitors (PARPi) in patients with ovarian cancer and HRD deficiency, several clinical trials have already been contacted for pancreatic cancer.

Olaparib represents the first PARPi with approval for this group of patients. PARPi are also under investigation either alone or in combination with chemotherapy or immunotherapy in patients with pancreatic cancer 9

It would be of interest if DNA methylation could potentiate the impact of DNA mutations in order to classify subgroups of patients who may benefit from this mode of treatment with remarkable efficacy.

BRCA1/2 mutations can increase the possibility of pancreatic ductal adenocarcinoma (PDAC), however, their impact on the clinical features of the disease is less clear. Multiple cohort studies have shown that PDAC patients with germline mutations such asBRCA1, BRCA2, PALB2, CDKN2A, and ATM, are diagnosed earlier with PDAC as compared to those without germline mutations 13, 15-17. On the other hand, their prognostic value remains controversial since several studies have shown mixed results. To date, in a large cohort study, including 71 BRCA-positive PDAC patients a median OS of 14 mo was found for the whole cohort and 12 mo for patients with stage 3/4 disease. At the time of publication, the median OS for early-stage disease had not been reached as 52% of patients were still alive at 60 mo 18. These findings suggest that BRCA-mutated PDAC patients may have a considerably better prognosis than that reported for the general PDAC population. More recent case-control studies reported by Blair et al 8 support the opposite results. PDAC patients with BRCA1 and BRCA2 mutations were compared to age-matched controls. The survival analysis has shown that both OS and disease-free survival (DFS) were lower in carriers than in controls. In another case-control study comparing BRCA mutation-positive, early-stage PDAC patients undergoing surgical resection to age-matched BRCA-wildtype controls, has shown no significant differences in median OS or DFS between the groups. The study concluded that BRCA mutations had a small prognostic impact in early PDAC 19.

The role of the epigenetic silencing of BRCA1/2 has been well described in breast and ovarian cancer, but in pancreatic cancer has yet to be elucidated. Maxwell et al. 20 reported BRCA1 locus-specific LOH in 37 of 41 (90%) gBRCA1 carriers diagnosed with breast cancer and in 48 of 52 (93%) patients with gBRCA1-ovarian tumors. In their sub-cohort of patients who underwent methylation-specific PCR, somatic promoter methylation was observed in eight of 23 (35%, range not reported) gBRCA1-breast tumors and three of 15 (20%, range not reported) gBRCA1-ovarian tumors. Many studies are needed to establish whether BRCA1/2 is epigenetically affected in pancreatic cancer and other tumor types.

In this study, we attempted tο detect and observe the methylation status of BRCA1/2 genes in the cell-free DNA of a small cohort of pancreatic cancer patients. More specifically, the study population consisted of 55 patients with operable disease and 50 patients with metastatic disease. In the operable setting BRCA1 was frequently found to be methylated and the methylation status was correlated with shorter overall survival. BRCA2 promoter methylation was associated with low-grade pancreatic cancer (p=0.023). Patients with methylated BRCA2 gene presented statistically shorter overall survival (OS) as compared to those with an unmethylated one (p=0.001). In the metastatic setting, methylation of BRCA1 and BRCA2 genes was also a frequent event affecting almost 50% of patients. BRCA2 promoter methylation was also correlated in this group of patients with low-grade pancreatic cancer (p=0.018). Only BRCA1 methylation was associated with shorter overall survival (OS).

These results represent one first remark that DNA methylation in BRCA1/2 could be an alternative way of gene silencing in pancreatic cancer patients. Nevertheless, we lack some useful information regarding the methylation status of the tissue sample or the presence or absence of the BRCA1/2 mutation. On the other hand, we obtained the DNA methylation profile of BRCA1/2 in the cell-free plasma DNA which needs to be confirmed in a larger cohort of patients in order to establish its clinical usefulness.

## Figures and Tables

**Table 1:** Clinical characteristics of the patients

Patient characteristics	All patients no.	Patients with oPC	Patients with mPC
**No. of patients**	105	55	50
**Gender**			
Males	70	41	29
Females	35	14	21
**Age, years**			
≤60	20	12	8
>60	85	43	42
**Grade**			
Well	5	4	1
Moderate	60	31	29
Poor	18	14	4
Unknown	22	6	16
**Histology**			
Adenocarcinoma	89	55	34
Unknown	16	0	16
**PS**			
0-1	101	54	47
2	4	1	3
**Stage**			
IIA		6	
IIB		14	
III		35	

## Abbreviations

MSP: methylation-specific PCR
OS: Overall Survival
PFS: Progression-free survival
